# Cytological features of pure micropapillary carcinoma of various organs: A report of eight cases

**DOI:** 10.3892/ol.2014.2198

**Published:** 2014-05-29

**Authors:** GÜLBEN ERDEM HUQ, ŞULE CANBERK, MELTEM ÖZNUR, PELIN YILDIZ, BURAK BAHADIR, KEMAL BEHZATOĞLU

**Affiliations:** 1 Department of Pathology, Istanbul Education and Research Hospital, Istanbul 34098, Turkey; 2 Department of Pathology, Istanbul University, Cerrahpasa Medical Faculty Istanbul 34320, Turkey; 3 Department of Pathology, Namık Kemal University Medical Faculty, Tekirdağ 59100, Turkey; 4 Department of Pathology, Bezmialem University, Medical Faculty, Istanbul 34093, Turkey; 5 Department of Pathology, Bülent Ecevit University, Zonguldak 67100, Turkey

**Keywords:** micropapillary carcinoma, cytology, parotid, lung, urinary bladder, breast

## Abstract

Micropapillary carcinoma (MPC) is a rare aggressive tumor, which generally accompanies the primary carcinoma of the organ of its origin, while the pure form is extremely uncommon. Angiolymphatic involvement is widespread and a considerable proportion of the cases present with metastases. The current study presents eight pure MPC cases arising from the breast (n=3), urinary bladder (n=3), parotid gland (n=1) and lung (n=1, presenting with pericardial effusion), with the cytological findings. The eight patients included three female and five male cases aged between 48 and 74 years. The most common cytological findings were three-dimensional aggregates, cell clusters with angulated or scalloped borders, single cells with a columnar configuration and eccentric nuclei, and high-grade nuclear features. Histopathological sections showed accompanying *in situ* ductal carcinoma in the cases of MPC arising in the parotid gland and breast (n=3), and one case in the bladder exhibited only *in situ* MPC. The average follow-up period was 20 months (range, 6–54 months) and, during this period, three patients succumbed to the disease. At present, four patients are alive with disease and one patient is alive and disease-free. In conclusion, cytology is an important tool for the diagnosis and management of MPC.

## Introduction

Micropapillary carcinoma (MPC) is an uncommon morphology typically observed within the context of the borderline serous carcinoma of the ovary (1). In addition, similar morphological characteristics of MPC are rarely detected in other non-ovarian sites, such as the breast, urinary bladder, lung, parotid glands and colon (2–7). In anatomical locations other than the ovaries, MPC exhibits an aggressive course and generally accompanies the primary neoplasm of the organ of its origin (2).

Histologically, MPC has a typical appearance, characterized by cells with eosinophilic cytoplasm that exhibits a nest-like arrangement in artifactual spaces. The appearance is reminiscent of enlarged angiolymphatic vessels and is associated with a more aggressive clinical course and a higher rate of lymph node metastasis compared with the typical carcinomas of the organ of origin. Regardless of the accompanying tumor, the majority of MPC must always be documented in the pathological diagnosis.

Although less well characterized than its histological features, the cytological features of MPC have also been well defined. This is predominantly due to the increasing use of fine needle aspiration (FNA) biopsy, particularly in the parenchymal organs, which allows earlier diagnosis of this aggressive tumor (8–10). The early diagnosis of MPC with FNA biopsy may also be useful in uncommon sites, such as the parotid gland and pericardium.

In parenchymal organs, such as the breast, lung and parotid glands, where FNA has a major diagnostic role, it is important to be aware of MPC as a disease entity and to document its presence. Special care must be provided when evaluating lymph node and serous surface aspirations, due to their high tendency toward angiolymphatic spread and metastasis.

The current study assessed the cytological characteristics and emphasized the diagnostic value of the cytological examination of MPC in a total of eight cases with histologically confirmed diagnoses of pure MPC occurring at different sites, including the breast, urinary bladder, pericardium and parotid gland. The patient provided written informed consent.

## Case report

### Introduction to cases

A total of eight cases evaluated at the Department of Pathology, Istanbul Education and Research Hospital (Istanbul, Turkey) between 2005 and 2012 were included. Of these, two were originally from other centers, but were referred to the Department of Pathology, Istanbul Education and Research Hospital for consultation (cases 1 and 2). All cytological and histological materials were reassessed by two of the authors.

The diagnosis of MPC was based on assessment according to the following criteria of the most common findings: Tight clusters; three-dimensional cell aggregates with high-grade nuclear features; formation of morula, cell balls and staghorn structures; and single cells with columnar configuration and eccentric nuclei.

### Case 1

A 74-year-old male presented to the clinic with a painful parotid mass. The FNA revealed malignant cytology, not otherwise specified (NOS). A right radical parotidectomy was performed. The tumor was 1.2 cm in diameter and exhibited irregular borders, and no tumor was identified in the surgical borders. The diagnosis of *in situ* ductal MPC and pure invasive MPC was determined, with a pathological stage of pT2. The patient did not accept additional treatment and, to date, has survived for 14 months*,* with neck lymph node metastases.

### Case 2

A 60-year-old-male presented to the clinic with breathing difficulties and tachycardia. Pericardial aspiration led to a diagnosis of malignant cytology, NOS, but differentiation between mesothelioma and MPC was not possible. Computed tomography revealed a 2-cm mass in the right lung and bronchoscopic biopsy was performed. The diagnosis of pure MPC was determined. The patient received two cycles of chemotherapy; however, widespread metastases were found 4 months following the diagnosis and, subsequently, the patient succumbed to the disease.

### Case 3

A 52-year-old female presented to the clinic with a painless mass and skin dimpling on the upper-outer quadrant of the right breast. Modified radical mastectomy and axillary dissection were performed. The tumor was 3.4 cm in diameter. The diagnosis of pure MPC was determined and showed widespread intralymphatic tumor thrombi. MPC metastasis was present in six axillary lymph nodes and the pathological stage was pT3A. A total of six cycles of chemotherapy and radiotherapy were administered. At present, the patient is alive 14 months following the mastectomy, but has bone metastasis.

### Case 4

A 48-year-old male presented to the clinic with a painless mass at the midline upper quadrant of the left breast. FNA with ultrasonography was performed and the diagnosis of malignant cytology, MPC was determined. Modified radical mastectomy and axillary dissection were performed. The tumor was 2 cm in diameter. The diagnosis of pure MPC was determined and widespread intralymphatic tumor thrombi were observed. MPC metastasis was present in seven axillary lymph nodes together with tumor invasion of the chest wall. The pathological stage was pT3B. A total of six cycles of chemotherapy and radiotherapy were administered. Currently, the patient is alive 18 months following the mastectomy, but has recurrence on the chest wall.

### Case 5

A 53-year-old female presented with a painless mass in the upper quadrant midline of the left breast. FNA with ultrasonography revealed malignant cytology, NOS. Modified radical mastectomy and axillary dissection were performed. The diagnosis of pure MPC was determined. The tumor was 1.1 cm in diameter and widespread intralymphatic tumor thrombi were observed. Micropapilloma carcinoma metastasis was present in eight axillary lymph nodes and the pathological stage was pT3B. The patient received chemotherapy and radiotherapy; however, widespread metastases in the body were later found. The patient succumbed to the disease 54 months later.

### Case 6

A 72-year-old male patient presented with a complaint of hematuria. Cystoscopy revealed a solid ulcerated mass of 1 cm in diameter on the right lateral wall and a washout cytology sample was obtained. The diagnosis of malignant cytology, high grade urothelial carcinoma was determined and the mass was excised. Histological evaluation revealed a pure MPC with widespread lymphatic invasion, with a pathological stage of pT2. Radical cystectomy was performed and the pathological stage was found to be pT3N1. The patient received chemotherapy for 6 months and, to date, is alive with pelvic recurrence.

### Case 7

A 61-year-old male patient presented with hematuria. The cell block and urine cytology revealed malignant cytology, high grade urothelial carcinoma. Cystoscopy showed a solid mass in the trigone of 1 cm in diameter, which was resected by transurethral resection. Histopathological evaluation revealed a pure MPC and the pathological stage was pT2. Radical cystectomy was performed and the pathological stage was found to be pT3N1. In addition, MPC and widespread lymphatic invasion were present. The patient received six cycles of chemotherapy, but was identified to have widespread metastases in the body. The patient succumbed to the disease 54 months following the cystectomy.

### Case 8

A 58-year-old male presented with hematuria. Cystoscopy showed a flat lesion of 1.5 cm in diameter on the right lateral wall and a washout cytology specimen was obtained. The diagnosis of malignant cytology, MPC was determined and the mass was excised. On histopathological evaluation, the diagnosis of *in situ* MPC was determined and six cycles of bacille Calmette-Guérin treatment were administered. To date, the patient is alive and has been without disease for 6 months.

### Results

The clinical and cytological findings are summarized in Tables I and II. Five cases were male and three were female; the age range was between 48 and 74 years, with a mean age of 60 years. The average follow-up period was 20 months (range, 6–54 months). During this period, three patients succumbed to the disease; currently, four patients are alive with disease, and one is disease-free.

The locations of the MPCs were as follows: Three in the breast, three in the bladder, one in the parotid and one in the lung (with pericardial effusion). The mean tumor diameter was 1.9 cm, ranging between 1.1 and 3.4 cm.

**Figure 1:**
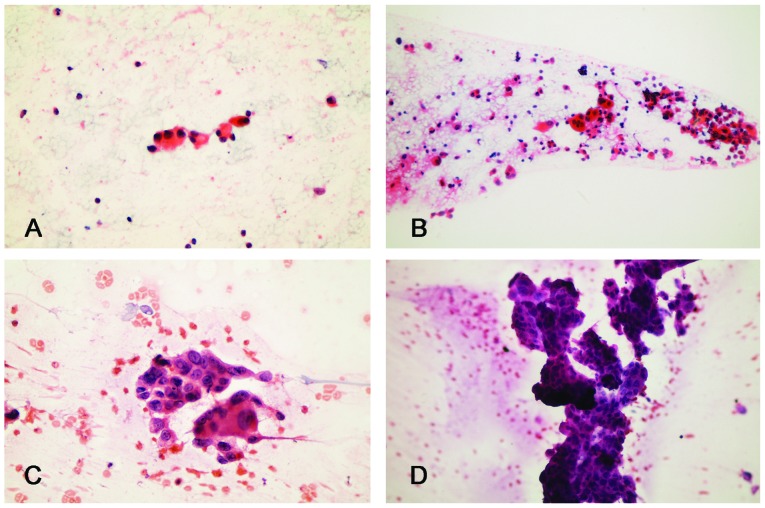
Cytopathology of micropapillary carcinoma samples obtained from different cases: (A) Case 5, single cells with columnar configuration and eccentric nuclei (stain, Papanicolaou; magnification, ×400); (B) case 4, cohesive tumor groups and scattered cells with apocrine-like cells (stain, Papanicolaou; magnification, ×200); (C) case 2, cohesive tumor groups and scattered cells with high-grade nuclear features indicating an invasive micropapillary component (stain, H&E; magnification, ×300); and (D) cohesive tumor cells forming large micropapillary structures indicating an *in situ* micropapillary ductal carcinoma (stain, H&E; magnification, ×200). H&E, hematoxylin and eosin.

**Figure 2:**
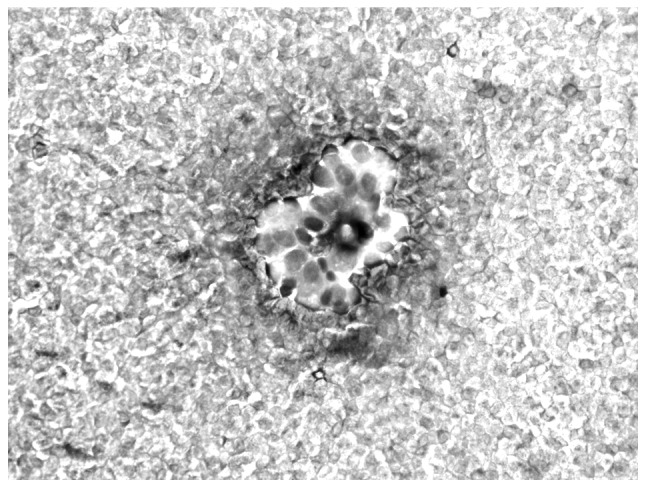
Case 1 revealed cohesive tumor cells forming a micropapillary cluster on a hemorrhagic background (stain, Papanicolaou; magnification, ×300).

Each case revealed different cytological MPC parameters; however, all parameters were detected in total. Three-dimensional aggregates, high-grade nuclear features, cell clusters with angulated or scalloped borders, and single cells with columnar configuration and eccentric nuclei were the most common findings (Figs. 1 and 2).

**Figure 3:**
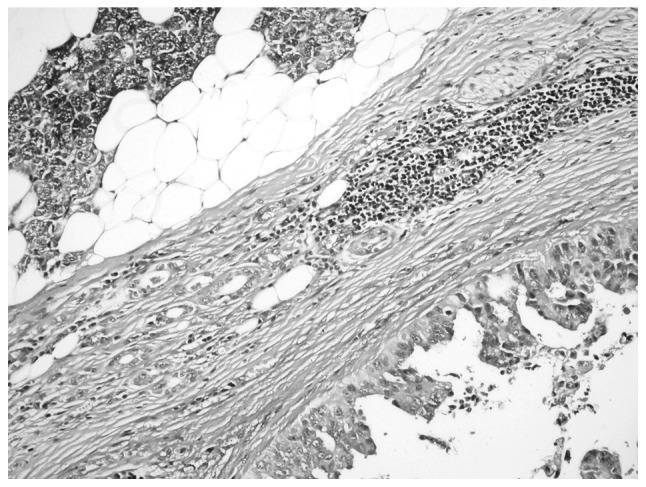
Case 1 revealed an *in situ* ductal carcinoma of micropapillary pattern in the parotid (stain, hematoxylin and eosin; magnification, ×200).

In seven cases, the diagnoses were confirmed by excisional biopsy; in one patient presenting with pericardial metastasis, confirmation was performed using bronchoscopic biopsy. A diagnosis of invasive pure MPC was determined in seven cases, accompanied by *in situ* ductal carcinoma in those patients with lesions in the breast and parotid gland (Fig. 3). In one case with MPC in the urinary bladder, only *in situ* MPC was present.

## Discussion

In addition to borderline serous carcinoma of the ovary, MPC has also been identified in other sites, such as the stomach, colon and parotid gland, over the last two decades (2–10). Despite its rarity, it deserves extra attention by virtue of its aggressive course. MPC coexisting with other morphological types of primary adenocarcinoma is more common than pure MPC. It may also initially present as distant metastasis due to a high rate of vascular invasion. The reverse polarity (inside-out growth) is the typical histological characteristic of the tumor that is likely to be essential in its pathogenesis. The direct contact between the secretory surface and the surrounding stroma is also likely to facilitate disease spread (2–10,12).

As emphasized previously, the early diagnosis of MPC is extremely important due to its aggressive course and, therefore, FNA and other cytological biopsy techniques have a major role in early diagnosis and management. FNA is a relatively easy biopsy method with a number of documented cases of breast MPC diagnosed using this approach (10,13,14). However, few cases of MPC in the urinary bladder have been reported, possibly due to the inapplicability of FNA and the availability of urine as the only cytological sample (15,16). Although an increased number of studies examining the role of cytological examination in the lung have been conducted compared with those in the urinary bladder (17), to the best of our knowledge, no cases of primary MPC in the parotid gland or MPC in the lungs presenting with pericardial findings have been previously reported.

MPC usually exhibits similar characteristics in all organs, which assists in the diagnosis. General cytological features of MPC are as follows: i) Background consists of mucin, tumor diathesis and particularly urine smear with a dirty background, including numerous inflammatory cells; ii) cell clusters include tight clusters, three-dimensional cell aggregates with high grade nuclear features, morula, cell balls, staghorn structures and cell clusters with scalloped borders; and iii) cytological findings comprise cells with a high nuclear/cytoplasmic ratio, dense cytoplasm, moderate to severe nuclear atypia, including some vacuolated cells and apocrine-like cells, scattered single cells with columnar configuration and eccentric nuclei

In the majority of the present eight cases, the aforementioned cytological characteristics were present. The most frequent cytological findings were three-dimensional aggregates with high-grade nuclear features, cell clusters with angulated or scalloped borders, and single cells with columnar configuration and eccentric nuclei. In addition, single apocrine-like cells were more frequent in the breast samples.

All of the cases had pure MPC and a malignant cytology was readily established. A diagnosis of malignancy is easier to establish than a direct diagnosis of MPC in cytological materials, particularly in the breast. The histological characteristics of the neoplasm accompanied by MPC and additional features, including the dirty mucinous background, marked nuclear pleomorphism, discohesive cell groups, single cells, and inability to observe the surrounding myoepithelial cells in the breast FNA, assist in a diagnosis of malignancy. However, a diagnosis of pure MPC may not be readily determined and requires a good knowledge of its cytology. MPC of the bladder is uncommon; studies describing its cytological features are rare (8,15,16).

Although its cytological features do not differ from those of MPCs in other organs, the differential diagnosis from high-grade urothelial carcinomas may be difficult. Three cases of bladder MPC in the current series had a diagnosis of malignant cytology; only the eighth case was determined as an MPC. The most distinguishing feature of this case was the sharp, distinctive borders of the cell clusters. This case was the micropapillary variant of *in situ* urothelial carcinoma. *In situ* MPC is less aggressive than its invasive counterpart; its course is similar to that of urothelial carcinoma and may progress (18).

Parotid aspiration in the present study showed characteristics of an *in situ* ductal carcinoma rather than the typical characteristics of invasive MPC. In addition, histological examination revealed a predominance of *in situ* ductal carcinoma (solid and micropapillary). The invasive component exclusively consisted of MPC. MPC in the parotid glands is extremely rare with only a few previous cases reported (6,19), and the current study presents the first report with cytological findings. In suspected cases of ductal adenocarcinoma of the parotid gland, the possibility of MPC must also be considered.

Furthermore, to the best of our knowledge, the presented case of lung MPC with initial pericardial presentation represents the first of such a case in the literature. As compared with the pleura, the pericardium is a less frequent site of metastasis. This patient with chest pain presented to Namık Kemal University Medical Center (Tekirdağ, Turkey) and a pericardial aspiration was performed. The microscopic slides were then referred to the Department of Pathology, Istanbul Education and Research Hospital for consultation. Initially, it was not possible to differentiate between mesothelioma and metastasis due to the cytological similarity between these two conditions. However, following the detection of a mass lesion in the radiological imaging studies, a biopsy was performed with a subsequent diagnosis of MPC. This suggests that MPC must be included in the differential diagnosis of less common metastasis sites, such as the pericardium.

It is important to recognize the well-defined cytological characteristics of MPC to determine a precise diagnosis. A malignant condition with high metastatic potential, such as MPC, may occur in extremely rare sites, including the parotid glands, or initially present on serous surfaces, such as the pericardium.

## Figures and Tables

**Table I:** Clinical findings.

Case, n	Age, years/Gender	Primer location	First clinical presentiation	Tumor size, cm/Stage^a^	Treatment	Disease outcome
1	74/M	Right parotid	Parotid mass	1.2/pT2	Radical paroidectomi	Alive with disease at 14 months
2	60/M	Lung	Pericardial	2/pT4	CTh	Mortality at four effusion months
3	52/F	Right breast	Breast mass	3.4/pT3	MRM, axillary dissection, CTh and RTh	Alive with disease at 14 months
4	48/F	Left breast	Breast mass	2/pT3	MRM, axillary dissection, CTh and RTh	Alive with recurrence at 18 months
5	53/F	Left breast	Breast mass	1.1/pT3	MRM, axillary dissection, CTh and RTh	Mortality at 54 months
6	72/M	Urinary bladder	Hematuria	1/pT3	Cystectomy and CTh	Alive with recurrence at eight months
7	61/M	Urinary bladder	Hematuria	2/pT3	Cystectomy and CTh	Mortality at 43 months
8	58/M	Urinary bladder	Hematuria	1.5/pTa	BCG	Alive at six months

a According to the American Joint Committee on Cancer staging system (2010) (11).

M, male; F, female; MRM, modified radical mastectomy; CTh, chemotherapy; RTh, radiotherapy; BCG, bacille Calmette-Guérin.

**Table II:** Cytological and histological findings of MPC.

		Cytological findings			
				
Case, n	Sampling method	Background and single cells	Cell clusters	Cytological diagnosis	Histological diagnosis
1	FNA	A few isolated high-grade malignant cells	Three-dimensional solid epithelial aggregates and monolayer sheets	Malignant cytology, NOS	Micropapillary carcinoma with *in situ* ductal carcinoma
2	Pericardial aspiration	Isolated malignant cells and high-grade nuclear features	Three-dimensional aggregates	Malignant cytology, NOS	Micropapillary carcinoma
3	FNA	Single cells with a columnar configuration and eccentric nuclei and high-grade nuclear features	Small cell groups	Malignant cytology and micropapillary carcinoma	Micropapillary carcinoma with *in situ* ductal
4	FNA	Isolated malignant cells, high-grade nuclear features and apocrine-like cells	Three-dimensional aggregates and small cell groups	Malignant cytology and micropapillary carcinoma	Micropapillary carcinoma with *in situ* ductal
5	FNA	Isolated malignant cells, and high-grade nuclear features	Small cell groups	Malignant cytology, NOS	Micropapillary carcinoma with *in situ* ductal
6	Urine (washout material)	Isolated malignant cells, high-grade nuclear features and eccentric nuclei	Three-dimensional aggregates and morula-like structures	Malignant cytology and urothelial carcinoma	Micropapillary carcinoma
7	Urine and cell block	Isolated malignant cells, high grade nuclear features and irregular nuclear contours	Cell clusters with angulated borders and scallopes	Malignant cytology and urothelial carcinoma	Micropapillary carcinoma
8	Urine (washout material)	Isolated malignant cells, high grade nuclear features and irregular nuclear contours	Cell clusters with angulated borders and scallopes	Malignant cytology and possible micropapillary carcinoma	*In situ* micropapillary carcinoma

MPC, micropapillary carcinoma; FNA, fine-needle aspiration; NOS, not otherwise specified.
